# Inaugural meeting of the Pan-American Society for Evolutionary Developmental Biology report: the importance of diversity in a multidisciplinary field

**DOI:** 10.1186/s13227-015-0035-1

**Published:** 2015-12-09

**Authors:** Allison Edgar, Javiera Chinga

**Affiliations:** Department of Biology, Duke University, Durham, NC 27708 USA; Department of Ecology, Faculty of Biological Sciences, Pontificia Universidad Católica de Chile (PUC), Alameda 340, Santiago, Chile

**Keywords:** Diversity, Interdisciplinary, Multidisciplinary

## Abstract

We analyze the interdisciplinary state of evolutionary developmental biology based on the diversity of themes, taxa, levels of organization and scientists at the first meeting of the Pan-American Society for Evolutionary Developmental Biology (2015). We first highlight selected presentations representative of three themes: gene regulatory control, developmental patterning mechanisms, and ecological-evolutionary-developmental interactions. We summarize the questions, approaches, and taxonomic sampling of plant and animal research presented at the meeting. Finally, we synthesize themes from the meeting’s panel discussion and workshops on broadening participation, education, and the role of Evolutionary Developmental Biology in the scientific community and its ability to transcend and integrate fields of inquiry.

## Background

Evolutionary Developmental Biology (Evo-Devo) examines how changes in developmental trajectories shape evolutionary novelty and diversity. To understand the relationship between development and evolution, we must integrate different levels of organization, from molecules to organisms to ecosystems. In this letter, we discuss the interdisciplinary nature of Evo-Devo through the lens of the inaugural meeting of the Pan-American Society for Evolutionary Developmental Biology (Pan-Am Evo-Devo) [1, 2]. The meeting was held at the Clark Kerr campus of the University of California at Berkeley, USA during August 5–9, 2015. The meeting was organized by outgoing society president Ehab Abouheif, Christopher J. Lowe, Nipam H. Patel, and incoming president Karen E. Sears, with additional local organizers Karen D. Crow and Chelsea D. Specht.

Presentations from a mixture of established, early-career, and student-stage scientists emphasized the diversity of questions, approaches, and systems used by this international Evo-Devo community, as well as the diverse views of the scientists themselves. Notably, the same mixture of career stages was also represented in the poster session, providing attendees the opportunity to visit posters presented by students, post-docs, and principal investigators alike. Presentations showcased contemporary Evolutionary Developmental Biology’s power to answer long-standing fundamental questions at a mechanistic level [3]. A forthcoming meeting report [4] and special issue of JEZB will consider the meeting from other perspectives. Here, we consider a few common research themes from the talks and panel discussion to address the following questions: What does the interdisciplinary Evo-Devo perspective uniquely offer biology as a whole? What interdisciplinary approaches are emerging in Evo-Devo? What future interdisciplinary challenges does the field face?

## Research themes

### Evolution of gene regulatory control

Re-deployment and modulation of developmental processes to generate novelty and selectable variation is a key area of inquiry for Evo-Devo [5–7]. Sean Carroll’s keynote (US)^1^ focused on co-option as a source of novelty. Moving from fly wings [8] to vertebrates, he reminded us that diversification of a complex phenotype (in this case, snake venom composition) does not require gene duplication, and may even result from gene loss. Co-option of core signaling pathways to novel contexts was a theme picked up by other speakers. Mark Rebeiz (US) examined the network changes involved in GRN co-option to form a novel genital structure in *D. melanogaster*, finding that an enhancer from a species without the structure can drive expression of the GRN circuit, which suggests that derived deployment need not create new constraints or discard ancestral functions. Cecilia Zumajo (Colombia/US) studied the evolution of AP2 in seed plants, showing that the A function in sepal and petal identity is not conserved outside rosids, while the less studied role in fruit development is likely conserved in all angiosperms. Alma Pineyro-Nelson (US/Mexico) showed that changes in the ABC model in petaloid monocots may be due to changes in the domain of action of UFO, a trans-regulator of AP3 and regulator of B or C gene function in diverse angiosperms [9].

Cis-regulatory element evolution, particularly how pleiotropic consequences of gene networks are buffered or even leveraged, is a mechanistic counterpart to co-option. Veronica Hinman (US) presented an example of a conserved primary and a diverged secondary binding motif for an echinoderm transcription factor, suggesting that novel low-affinity motifs modulate its protein level and contribute to its functional modularity. Ralf Sommer’s (Germany) was a tale of complex regulatory changes controlling an ecologically responsive dimorphism: a nematode that has co-opted the autocrine signaling used to induce the dauer phenotype familiar from *C. elegans* to produce a carnivorous morph [10]. The switch gene [11] itself is regulated by chromatin remodeling at multiple independent levels, including an embedded antisense message in the switch gene. Bob Reed (US) revealed how modular enhancer elements map beautifully onto butterfly wing pattern elements, allowing one transcription factor to control multiple spatial patterns, and also discussed an ecologically relevant pattern dimorphism [12].

### Patterning and polarity across scales

Conservation, change, and convergence of individual genes, gene network logic, and signaling pathways are the mechanistic interface of evolution and development. Below, we consider several presentations that examine these phenomena in patterning at different scales.

#### Cells and tissues

Patterning and polarity of cells and tissues came up across different taxa and developmental stages alike. Two such talks specifically investigated the origins of multicellularity. Matt Gibson (US) asked: “What is the ultimate animal shared derived trait?” The answer, of course, is polarized epithelial sheets. Gibson discussed his recent work on cell polarization in Nematostella cleavage, positing that re-instatement of apical-basal polarity is required for normal blastoderm formation. Mariana Benítez (México) applied a Dynamical Patterning Modules (DPMs) approach to the study of cell differentiation patterns in the transition to multicellularity with emphasis on plants, showing that different types of communication among cells generate different, robust multicellular patterns in which cells not only aggregate, but also begin differentiation [13, 14].

#### Organs

Most of the talks on plants were about organ-level patterning. The Early Career Award lecture delivered by Natalia Pabón-Mora (Colombia) revealed gene regulatory network evolution underlying floral and fruit diversity (and also modeled the kind of accessible communication that builds bridges by using analogous animal groups to orient those less familiar with plant evolutionary relationships) [15, 16]. Neelima Sinha’s keynote (US) examined gene regulatory network modules controlling intra-specific diversity in solanum leaf shape, showing that rather than modifying the distribution of the plant hormone auxin, different leaf shapes arise by modulating maturation time of individual leaves, changing the leaf’s window of sensitivity to the signal [17]. Jocelyn Hall (Canada) reported that although the TCP family of transcription factors related to the convergent evolution of monosymmetry in flowers had undergone a heterochronic shift in Brassicaceae, virus-induced silencing of these genes in Cleomaceae shows a role in petal identity but does not account for the contrasting symmetry between Cleomaceae and Brassicaceae [18]. At the cellular level, Angela Hay (Germany) described the morphomechanical innovation of the *Cardamine hirsute* seed valve: a bilayer with higher lignin content in the inner layer, resulting in cell geometry and anisotropy that allows turgidity-induced cell shape changes to generate force for explosive seed dispersion. Important taxonomic contributions to understanding of the evolution of the floral organ identity genes and their consequences for flower morphology included Harold Suárez-Baron (Colombia), on flower and perianth development in the basal angiosperm *Aristolochia fimbriata*.

#### Bauplan

Body patterning, including symmetry, and the many functions of the Hox genes were, as ever, a favorite topic. Rich Palmer (Canada) discussed left–right asymmetry in organisms diverse as plants and arthropods; his talk was the only one to synthesize underlying mechanisms in plants and animals. He suggested that the prevalence of transitions from random (dimorphic, left and right) to fixed (left or right) asymmetries is an example of genetic control evolving after the fact to canalize a developmental phenotype [19]. Karen Crow (US) showed collinear HoxA expression in the hind-gut and vent of ray-finned fishes, as well as HoxA/D expression in a novel paddlefish feature of that occurs in an anterior domain that was previously considered a “hox-free” region of the head in a paddlefish novel outgrowth [20]. Paul Gonzalez (US) has been working on an indirect-developing hemichordate to compare its regional specification to that of the direct-developer *Saccoglossus kowalevskii*. An Evo-Devo knowledge gap in whole-body regeneration was addressed by two presentations. Mansi Srivastava (US) reported that regeneration in an acoel flatworm model she is developing suggests that regenerative mechanisms known from planarians may be ancient to all metazoans [21]. Alexa Bely (US) showed time-lapse imaging of the regeneration process documenting neoblast behavior, and raised questions about cell lineage in annelid regeneration [22]. Finally, Vivian Irish (US) used a citrus model [23] to show that thorns possess meristematic characteristics but differentiate when forced out of the stem cell niche. Stacy D. Smith (US) showed that although convergent losses of purple pigmentation in Iochrominae (Solanaceae) are possible through structural mutations in the pigment pathway (e.g., loss of enzyme function), fixed transitions to white flowers are largely due to changes in gene expression [24–26].

### Ecological inputs and consequences

Rudy Raff (US) delivered the society’s first Pioneer Award Lecture, tying many of the meeting’s themes back to a common ancestor: his work on rapidly evolving life-history transitions among sea urchins using hybridization of direct and indirect-developing species [27]. Raff also discussed the results of his experimental investigations into fossilization of marine embryos, showing that under certain conditions common marine microbes can replace cells with a biofilm community that is itself susceptible to fossilization [28]. We usually think of development as largely screened off from selection by the adult phenotype produced, but Matt Rockman (US) examined what happens when selection operates during development, as in the evolution of direct (lecithotrophic) from indirect (planktotrophic) development in a polychaete [29]. Sofia Casasa (US) talked about how manipulating components of the insulin pathway, insulin receptor, and transcription factor FoxO, alters food-responsive allometry curves in beetle horns. Catherine Linnen (US) showed that changes in host use among pine sawflies generate barriers to gene flow, in one of the few presentations to measure an ecological output.

In plant talks the role of ecological conditions was always implicit, from the regulatory role of shade on the degree of compoundness in solanum leaves, to the role of floral symmetry, color, and key morphological innovations on pollination, to seed dispersion mechanisms. This general attention to the environmental-organism relationship may result from the sessile nature of plants, which have to deal with environmental changes in a single location.

## Study systems

Broad sampling of taxonomic groups [30–32] and levels of organization [33] is crucial to understanding novelty and diversity. Comparisons across both great and small taxonomic distances were represented at the meeting. Attendees widely expressed enjoyment that the meeting was based on shared theoretical background and informed by a wide range of organisms and approaches. Scientists met who, by virtue of their study species’ divergence, would not have encountered one another at other meetings they regularly attend. Many also expressed the hope that even more diverse research approaches, including dynamic systems theory, paleontology, and ecology, will see increasing representation at future meetings.

**Fig. 1:**
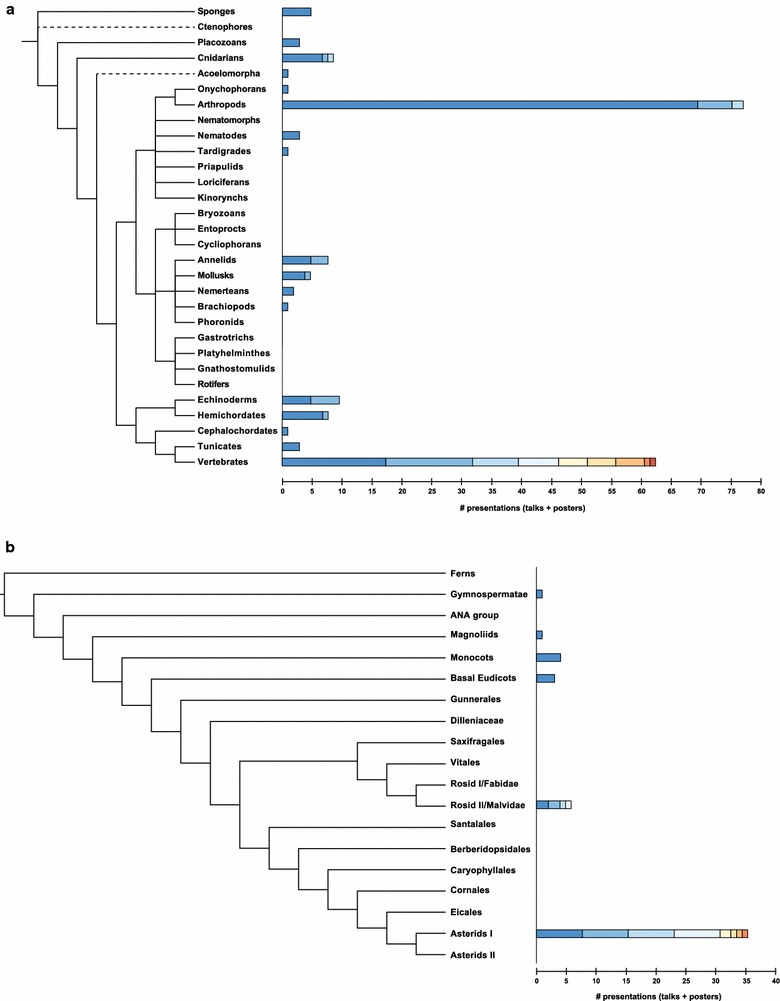
Taxonomic representation among presentations at the Pan-American Society for Evolutionary Developmental Biology 2015 meeting. 57 oral presentation and 164 of 179 published poster abstracts are included in the analysis; of excluded papers seven did not consider particular organisms (theory, modeling, history of science), three were comparisons at a higher taxonomic level, two focused on organisms outside these groups, and three were withdrawn or absent from the meeting. Comparative research using multiple taxa was counted for each group. **a** Stacked bar graphs at terminal branches (metazoan phyla) show the number of combined oral and poster presentations using each group. *Each colored bar segment* represents a different class within the named phylum in *descending order* of frequency (*listed alphabetically* in case of equal numbers). Presentations containing data from multiple taxa are counted for each group represented. Sponges (Demospongiae), Placozoans, Cnidarians (Anthozoa, Hydrozoa, Schyphozoa), Acoelomorpha (Acoela), Onychophorans (Udeonychophora), Arthropods (Insecta, Crustacea, Arachnida), Nematodes (Chromadorea), Tardigrades (Eutardigrada), Annelids (Polychaeta, Clitellata), Molluscs (Gastropoda, Cephalopoda), Nemerteans (Holopnemertea), Brachiopods (Lingulata), Echinoderms (Actinaria/Echinoidea), Hemichordates (Enteropneusta, Pterobranchia), Cephalochordates (Branchiostomiformes), Tunicates (Pleurogona), Vertebrates (Actinopterygii, Mammalia, Amphibia, Reptilia, Aves/Chrondrichthyes/Hyperoartia, Myxini). **b** Stacked bar graphs at terminal branches show the number of combined oral and poster presentations for vascular plant taxa. *Each colored bar segment* represents a different genus in *descending order* of frequency (*listed alphabetically* in case of equal numbers). Gymnospermatae, Magnoliids (Aristolchia), Monocots (Erythonium/Pooidae/Zingiberales), Basal Eudicots (Aquilegia), Rosiid II/Malvidae (Arabidopsis/Cardamine, Cleome, Citrus), Asterids I (Jaltomata/Lochrominae/Petunia/Schizanthus/Solanum)

Even so, interdisciplinary approaches at the conceptual level do not necessarily produce an even distribution of model systems at the taxonomic level. Of 57 talks, 44 (77 %) studied animals, 11 (19 %) plants, and one (1.7 %) algae. There are logistical, historical, financial, and geographic constraints on taxonomic representation, but it is a worthy goal to examine development across and within independent origins of multicellularity. The best-represented animal groups were fishes (predominately ray-finned fishes) and insects (mainly dipterans and coleopterans), together making up nearly half of the zoological talks, while the remainder was widely distributed across Metazoa. Figure 1a shows the distribution of sampling across animal phyla in both talks and poster presentations, estimated from published abstracts. The 25 total oral and poster presentations about plants represented 13 angiosperm families (Fig. 1b), all with an explicitly comparative approach. However, only one talk considered other seed plants. The family with the most comparisons at the species and genus level was Solanaceae. As a point of comparison, the Euro Evo Devo (EED) 2014 meeting (Vienna) [34] had 219 talks and of these 163 (74 %) studied animals, 25 (11 %) plants, one (0.5 %) algae and one (0.5 %) virus, showing that animal models remain characteristic of the field. The EED meeting had higher number of plant taxa studied than the Pan-American meeting, which may be a consequence of the meeting’s larger size.

As for the levels of organization studied, zoological research ranged widely across levels—whole body, complex morphological units, single organs, simple tissues, and single cells. In contrast, almost all studies of plants were at the organ level: leaves, flowers or fruits, usually comparing developmental genetic findings between and within organs at different taxonomic levels. The modular nature of plant development, with its high incidence of homeotic transformation within organs, makes Evo-Devo concepts (and therefore studies) more tractable at the organ level, which may explain this trend. Complex phenomena beyond morphology, particularly those with ecological consequences, have long been a key interest for animal Evo-Devo. However, the outcomes of interest are often primarily developmental (e.g., life-history transitions, environmental polyphenisms); explicit studies of ecological outcomes are needed to complete the picture.

## Interdisciplinary approach

Development translates genotype into phenotype, and so the evolution of this process naturally touches on many aspects of biology. Evo-Devo studies have long drawn from diverse approaches including embryology, morphology, paleontology, ecology, developmental genetics, and gene networks [33, 35–38]. Integration of knowledge is a major goal of contemporary life science, and interdisciplinary research has great potential for long-term impact [39]. However, multidisciplinary integration does not emerge spontaneously as bodies of knowledge grow to touch one another. It is necessary to build shared technical language among scientists from diverse backgrounds to generate new hypotheses and extend understanding of phenomena across systems and levels of biological organization [40].

**Fig. 2:**
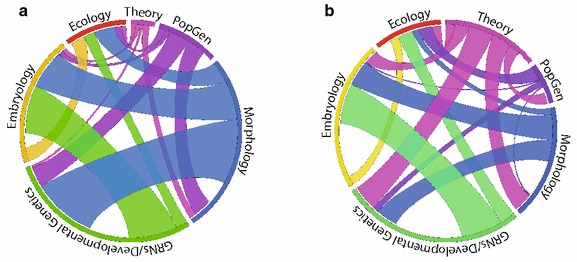
Methodological intersections in oral presentations displayed as Circos diagrams [41] using the ratio layout function to visualize interdisciplinarity. We consider the methods of six disciplines, defined to be independent within the sample set (e.g., paleontology was excluded from the visualization although it is included in the text because all paleontology talks included morphology) and such that both animals and plants are amenable to the techniques. (1) Morphology, meaning explicit analysis of a non-binary size or shape output (*blue*); (2) gene regulatory network (GRN) analysis and developmental genetics (*green*); (3) experimental or comparative embryology (*yellow*); (4) ecology, meaning measurement of an ecological variable (*red*); (5) theory, including mathematical modeling (*pink*); (6) population genetics and QTL analysis (*purple*). **a** All oral presentations from the Pan-American Society for Evolutionary Developmental Biology 2015 meeting are included, scored from published abstracts and our own notes from the talks. **b** All oral presentations from the Euro Evo Devo 2014 meeting, scored from published abstracts only, using the same analytical framework and color scheme as **a**

Of the 57 talks, 48 (84 %) used developmental genetics or gene regulatory network (GRN) analysis, 27 (47 %) examined morphology, 25 (44 %) used comparative embryology, and three (5 %) focused on paleontology. While 20 (18 %) talks highlighted the ecological significance of developmental phenomena, only nine (16 %) explicitly applied ecological concepts. Only four talks used modeling as a primary method. Multidisciplinary methodology was the rule: 42 talks (74 %) applied two or more, 23 (40 %) applied three or more, and eight (14 %) applied four or more of the above disciplines’ methods. As a point of comparison, we analyzed the published abstracts from the EED 2014 meeting using the same framework. Since we did not attend that meeting the coding may be more error-prone, but we found generally concordant patterns between the two conferences. Developmental genetics and GRN analysis was most common (65 % of 219 talks), followed by embryology (29 %) and morphology (26 %). Paleontology was similarly less studied (8 %). We found that a higher percentage of talks at the EED meeting included mathematical modeling (23 vs 7 %), which we attribute to the EED meeting’s inclusion of a special section dedicated to physical forces in development, and a lower percentage of used population genetics (5 vs 18 %). It is noteworthy that the percentage of talks considering ecology was similar between the two meetings (11 vs 16 %) even though the EED meeting had special sections for this category. Figure 2 highlights intersecting use of six methodological categories across all oral presentations as Circos diagrams [41].

Formal workshops and discussions ranged from technical advice for bringing cutting-edge tools to emerging systems to the role of modeling to education to broadening participation. In a panel discussion, “The Future of Evo-Devo,” one of the principal questions was how to better foster collaborative work. The interdisciplinary nature of Evo-Devo is its great strength; many in the panel discussion—panelists and audience—shared this view. Lena Hileman (US) summarized this view, saying that Evo-Devo’s focus on how multicellular diversity is generated and constrained by development can contribute to, learn from, and integrate disparate fields. Trisha Wittkopp (US) added that new ways of dissecting traits, including tests for selection in non-coding regions of the genome, will only continue to increase Evo-Devo’s intersection with population genetics as we learn more about how development generates selectable variation. For example, asking how development influences fitness and selection at the population genetic level complements studies examining gene regulatory control of ontogenetic trajectories. Moreover, the importance of ecology to Evo-Devo is increasingly apparent [38, 42, 43] and was broadly discussed, underlining that multilevel studies and broad taxonomic sampling are also necessary to understand how the organism-environment relationship shapes evolutionary and developmental processes. The meeting itself exemplified both the benefits and challenges of cross-discipline communication. We come from diverse backgrounds and we are still working out a shared language to discuss our science. This is a challenge that all interdisciplinary work faces. Perfect communication faltered at times, through for two very different reasons: non-congruent definitions of key terms used differently across sub-fields, or a lack of faith that the audience already appreciates the underlying scientific motivations and is eager to hear the data.

## Science and society

“The Future of Evo-Devo” panel discussion launched a passionate debate about community standards belonging to the zeitgeist: the excitement of the genome editing age, profound anxieties over looming anthropogenic environmental changes and our world’s severe resource inequality, and gnawing worry about shrinking funds for basic research. The debate was sparked by Heather Bruce (US), who asked whether Evo-Devo biologists should “make” animals not found in nature, a question with possible applications for basic research into extinct taxa [44] or conservation and restoration of endangered species [45], along with serving the fantasies of the privileged (e.g., designer pets). Cassandra Extavour (US) advocated caution, making sure that we apply the powerful tools at our disposal to worthy questions. The discussion acknowledged the need to ensure that ever-thinner research budgets are actually applied to projects that balance feasible approaches with questions truly worth answering. While the ethics of how to choose these questions and approaches will never be straightforward, it is important that we continue the conversation.

The acceptance of a research program is influenced by the historical and cultural background of the scientific community, just as scientific discovery can change the social vision of a phenomenon such as evolution [46]. Historical and cultural background influences the choice to pursue one set of questions or evidence over many others for good or ill e.g., [47]. One author has argued that, just as Charles Darwin’s thinking about selection was influenced by economic theory, E. E. Just’s milieu (the Black intellectual community of his time) contributed to his model of cytoplasmic-nuclear interactions in the egg [48]. For these reasons, we suggest that increasing diversity and multidisciplinarity may be parallel social processes. If this is the case, diverse identities of scientists themselves are not merely incidental to science but play a role in formulating creative research programs.

Attracting diverse people to the practice and appreciation of science was a key discussion point throughout the meeting. Evo-Devo’s interdisciplinarity appeals to synthetic and visual thinkers who might not otherwise see themselves as scientists, and is thus suited to introductory biology courses [49–51]. Students at all levels can be ambassadors to non-scientists in their larger communities, which is all the more reason to consciously include people from diverse backgrounds and perspectives. This meeting demonstrated the new society’s intention to be inclusive, arranging child-care, and holding workshops as a first step to greater participation and mentoring opportunities for career scientists of under-represented ethnicities, genders, national origins, sexual identities, and ability statuses. However, international representation showed room for improvement: of 57 talks, 41 authors were based in the US, seven were from Latin American universities (in Brazil, Chile, Colombia and México), three were from Canada, and six from countries outside the Americas (Germany, UK, Taiwan, Japan and Holland). Since abstract talks were awarded in proportion to the number of applicants from a given region, logistical and financial constraints on travel likely impacted who was able to attend. We saw a similar pattern in the EED meeting attendance, which reflected the convenience and cost of travel to the meeting location. We attempt no analysis of national origin or cultural identity. As we argue above, increased geographic representation can attract not only the biological diversity of local study systems but also diversity of thinking—new systems along with new ways of asking questions—and the Pan-American identity of the society can make an important contribution to scientific interaction across borders.

## Conclusions and future challenges

The Pan-Am Evo-Devo meeting was particularly broad in biological and methodological diversity across the talks. A common interest in development as a process of translating genotype to phenotype making changes in ontogenetic trajectories the basis of evolutionary change, allowed multidisciplinary approaches to stand out. Effective integration of a multi-level process—genetic background, gene regulatory interactions, cell behaviors, morphomechanics, ecological inputs and consequences, and more—is in its beginning and requires a broader diversity of models systems to produce a generalizable understanding of the relationship between evolution and development. Multilevel studies and broad taxonomic sampling are also necessary to understand how the organism-environment relationship shapes evolutionary and developmental processes.

We must become multilingual, speaking fluently human languages along with the formal and technical languages of developmental biology, evolutionary genetics, ecology, biophysics, paleontology, mathematics, computer science, and more to facilitate communication about our science and its ramifications in the world with colleagues, funding agencies, students, and the general public. Skilled ambassadors can reach across the bench or down the hall to colleagues to share how their work can benefit from an Evo-Devo perspective—or even let them know they are already practicing Evolutionary Developmental Biologists.

## Abbreviations

EDD: Euro Evo Devo
Evo-Devo: Evolutionary Developmental Biology
GRN: gene regulatory network(s)

## Footnotes

1 We parenthetically note participants’ current nation of work throughout the text.
